# Effects of spawning habitat on the performance of age-0 pumpkinseed sunfish (*Lepomis gibbosus*) ecotypes in a Canadian shield lake

**DOI:** 10.1007/s10641-026-01836-6

**Published:** 2026-03-28

**Authors:** Patrick Sobchak, Scott F. Colborne, Beren W. Robinson

**Affiliations:** 1 Department of Integrative Biology, University of Guelph, 50 Stone Road E., Guelph, ON N1G 2W1 Canada; 2 Great Lakes Institute for Environmental Research, University of Windsor, Windsor, ON N9C 1A2 Canada; 3 Quantitative Fisheries Center, Department of Fisheries and Wildlife, Michigan State University, East Lansing, MI USA

**Keywords:** Juvenile performance, Stable isotopes, Polymorphism, Ecotypes, Ecological opportunity, Adaptive radiation

## Abstract

Diversification between littoral and pelagic habitats is widespread in the fishes adaptively radiating in lakes, suggesting that where it occurs offspring spawned in a non-ancestral habitat may face few negative effects. We used littoral and pelagic ecotypes of pumpkinseed sunfish (*Lepomis gibbosus*) to investigate performance over the natal summer of age-0 juveniles spawned either in the ancestral littoral habitat of a Canadian shield lake or in two other habitat types: exposed rocky littoral shorelines and submerged rocky shoals offshore in the pelagic habitat. We compared zooplankton availability and use through stable isotope analysis of juvenile tissue and subsequent effects on late natal summer size and condition among habitats. Zooplankton prey were three times more abundant at pelagic than at littoral and shoreline sites and stable isotope analysis revealed that the fraction of plankton-derived tissue was consistently higher in age-0 juvenile fish from pelagic sites. By summer’s end, age-0 pelagic juveniles were 15% longer and 35% heavier than littoral and shoreline juveniles, indicating that accessible planktonic resources provided significant growth benefits to juveniles in the pelagic but not inshore habitats. By addressing a key uncertainty about the effects of non-ancestral natal conditions on age-0 performance, our study reveals a previously unrecognized juvenile benefit of habitat diversification by pumpkinseed sunfish in a postglacial lake. Since larger juvenile size contributes to first year survival these benefits could enhance local recruitment to a pelagic subpopulation that favours ecological and phenotypic diversification of pumpkinseed ecotypes through interacting developmental and evolutionary mechanisms.

**Supplementary Information:**

The online version contains supplementary material available at 10.1007/s10641-026-01836-6.

## Introduction

Colonization of non-ancestral conditions is one of several ‘ecological opportunity’ mechanisms (Yoder et al. 2010; Stroud and Losos 2016) that can launch adaptive diversification in animals (Schluter 2000; Nosil 2012; Wellborn and Langerhans 2014) including in fishes (Burress and Tan 2017). Studies of this process generally focus on adults, while largely overlooking the roles of early life stages. Juvenile stages may regulate several responses to non-ancestral conditions, such as demographic viability of a local subpopulation, population structure and connectivity (absent geographical isolation), and evolutionary responses by juvenile traits to diversifying selection. Consider the rapid adaptive divergence of the apple maggot fly (*Rhagoletis pomonella*) from an ancestral (native) hawthorn fly in North America (Rosser et al. 2022). Populations formed on the introduced European apple host increased the ancestral *Rhagoletis* population size and so demonstrated that the new host provided an ecological opportunity. Robust juvenile development on the novel host also generates adult host preference that drives host-specific mating and so decreases connectivity across different host fruits. Diversifying selection across host fruits favouring delayed larval diapause on apples relative to native fruits separates adult emergence times and so further strengthens reproductive isolation among host fruit populations (Filchak et al. 2000). Evolutionary responses to diversifying selection that decrease connectivity between viable subpopulations under different ecological conditions promote adaptive divergence by reducing genetic load (Malinsky et al. 2015). However, the roles of juveniles in population viability and connectivity, or as life stages under selection, have received less attention.

In fishes, adaptive radiation frequently involves shifts in habitat, yet the effects of different conditions on reproduction and on juvenile performance are rarely known. There are several reasons to expect juvenile life stages can play important roles. For example, larval and juvenile fish resource use influence condition, growth, survival, recruitment, and local population dynamics (e.g., Miller et al. 1988; Houde 1994; Nunn et al. 2012). Cannibalism and intercohort competition drives the relative dominance of juvenile and adult Eurasian perch (*Perca fluviatilis*) in a rare exploration of population dynamics in a trophic polymorphism (Svanbäck and Persson 2004). Plastic responses to local conditions that generate adult habitat preferences can isolate or connect populations (Turko and Rossi 2022). Plastic developmental responses to local conditions can influence foraging performance (Parsons and Robinson 2007), components of fitness such as growth (Robinson et al. 1996), and evolutionary responses to habitat conditions (Wimberger 1994; Robinson 2013; Parsons et al. 2016; Skúlason et al. 2019), in addition to less flexible larval traits that are targets of selection (Barrett et al. 2008; Marchinko 2009). Habitat is thought as reliably generating diversifying selection in a variety of lake fishes, including North American threespine stickleback, *Gasterosteus aculeatus* Linnaeus, 1758 (Schluter and Mcphail 1992; McKinnon and Rundle 2002), European whitefish (*Coregonus* spp., Doenz et al. 2018; Häkli et al. 2018), and African cichlids (*Cichlidea* spp., Seehausen and Wagner 2014), although the typical focus remains on adult performance (but see Skúlason et al. 2019).

Here we compare the influence of ancestral and non-ancestral habitats on performance in the first (natal) summer in larval and juvenile pumpkinseed sunfish, *Lepomis gibbosus* (Linnaeus, 1758), that underwent a habitat shift in northeastern North American post-glacial lakes (Robinson et al. 1993). We use ‘ancestral’ and ‘non-ancestral’ to describe the pre- and post-shift habitats respectively to highlight that whether a shift represents an ecological opportunity depends on the functional interaction of a focal species’ traits under different conditions from which they have likely recently evolved. Following a habitat shift, individual performance may be as good as under ancestral conditions because existing traits function well in both habitats. Near optimal growth, performance and survival of locally produced juveniles, in turn, should enhance local recruitment that increases the viability of a subpopulation in the non-ancestral habitat. In contrast, maladaptive ancestral traits in the non-ancestral habitat should limit reproduction and juvenile performance that, in turn, hampers local recruitment under non-ancestral conditions. Without sustained local reproduction and recruitment there is no opportunity for a viable ‘subpopulation’ in the non-ancestral habitat. Only persistent immigration can maintain individuals in such an ‘ecological sink’ situation. We compare juvenile performance between an ancestral and a non-ancestral (post-shift) habitat to evaluate effects on juvenile performance and consider the consequences for the viability of a local subpopulation.

A key challenge to evaluating the initial effects of a habitat shift on juvenile performance following a shift is ruling out the effects of subsequent evolutionary specialization that improves performance (Futuyma and Moreno 1988). Species comparisons overestimate performance because strong local adaptive responses are especially possible under reproductive isolation. Comparing groups connected by gene flow reduces this risk. Phenotypically and ecologically divergent ecotypes that coexist in numerous post-glacial lake fishes across littoral, pelagic and benthic habitats provide opportunities to compare ecotype performance in the face of gene flow (Robinson et al. 1993; Robinson and Wilson 1994; Snorrason et al. 1994; Schluter 1996; Bernatchez and Wilson 1998; Siwertsson et al. 2010; Seehausen and Wagner 2014; Oke et al. 2017; Skúlason et al. 2019; Tidy et al. 2023). Little consistent distinction exists between polyphenism and polymorphism in the literature (West-Eberhard 2003); nevertheless, we use polyphenism to refer generally to such intraspecific diversity to highlight that traits are frequently influenced by strong plastic developmental responses to local conditions, and that heritable elements of plastic developmental responses are involved in adaptive ecotype divergence in fishes (e.g., Skúlason et al. 2019). Polyphenisms in lake fishes range from continuous ecoclines to discrete ecotypes, suggesting varying degrees of local adaptation regulated by selection intensity and gene flow (e.g., Johannesson et al. 2025). Nevertheless, trophic traits are frequently less phenotypically divergent than the same traits between species (e.g., Foster et al. 1998; Riopel et al. 2008), consistent with a more recent origin. For these reasons, performance comparisons of recently originated ecotypes with limited genetic divergence reduces, but does not eliminate (e.g., Ford et al. 2015), the risk of biases in performance due to strong local adaptation.

We evaluate, using a well-studied trophic polyphenism (described below), the consequences of ancestral littoral and derived pelagic conditions on the performance of pumpkinseed larvae and age-0 juveniles over their natal summer. Conditions on non-ancestral pelagic shoals may benefit age-0 pumpkinseed spawned there because abundant zooplankton resources create an ‘ecological opportunity’ not just for adults but also for juveniles. Alternatively, competition from a high density of pumpkinseeds at pelagic sites (Jarvis et al. 2020) may limit juvenile resources and performance that constrains local recruitment there. A pelagic ecological opportunity hypothesis predicts: (i) a greater availability of zooplankton resources at pelagic compared to littoral sites; (ii) a greater contribution of planktonic source energy and nutrients to tissues in pelagic age-0 juveniles; and (iii) equal or greater growth and body condition in pelagic compared to littoral juveniles over the natal summer. Improved juvenile performance with access to pelagic zooplankton resources here strongly suggests that pelagic shoals provide an ecological opportunity to pelagic age-0 juveniles and adults. By promoting local recruitment to pelagic sites this benefit should favour pumpkinseed polyphenism.

## Materials and methods

### Pumpkinseed polyphenism

Pumpkinseed sunfish are a warm-water species that colonized postglacial lakes as they warmed in the northeastern portion of their range in North America. In relatively depauperate lakes, a littoral ecotype that consumes aquatic insect larvae and snails from the inshore benthos coexists with a pelagic ecotype that consumes larger Cladocera spp. zooplankton from submerged shoals in the offshore pelagic habitat (Robinson et al. 1993; Gillespie and Fox 2003; Jastrebski and Robinson 2004; Berchtold et al. 2015; Colborne et al. 2016). The pelagic ecotype is derived from the local littoral ecotype (Weese et al. 2012). The littoral ecotype is ancestral by virtue of specialized pharyngeal jaw traits that help to crush armoured littoral invertebrates such as snails (Huckins 1997) and other littoral prey that dominate the diet throughout their ancestral southern range (Scott and Crossman 1998). In postglacial lakes, competition for relatively poor littoral prey is thought to have driven a shift to feeding on zooplankton prey in the more productive pelagic lake habitat (Robinson et al. 2000; Weese et al. 2012). Occupying pelagic rocky shoals and islets, the pelagic ecotype has smaller oral and pharyngeal jaws (Berchtold et al. 2015), less widely spaced gill rakers, a smaller and shallower head and body shape, and smaller brains than the littoral ecotype (Robinson et al. 1993, 2000; Jastrebski and Robinson 2004; Gillespie and Fox 2003; McCairns and Fox 2004; Weese et al. 2012; Berchtold et al 2015; Colborne et al. 2016; Axelrod et al. 2018). Phenotypic plasticity contributes strongly to ecotype differences, but genetic differences in plastic developmental responses to feeding and habitat cues also distinguish ecotypes that appear adaptive (Robinson and Wilson 1996; Parsons and Robinson 2006; Januszkiewicz and Robinson 2007; Axelrod et al. 2022). Trade-offs in feeding performance between littoral and pelagic zooplankton prey (Parsons and Robinson 2007) and growth, especially for pelagic individuals (Robinson et al. 1996), likely generate selection on resource traits that can drive local adaptation to pelagic conditions. Nevertheless, adaptive responses may be limited for several reasons. Pumpkinseed likely delayed colonization of these lakes formed 12,000 years ago until they warmed sufficiently to meet their thermal preferences (e.g., Campana et al. 2020), suggesting a more recent habitat shift that limits adaptive responses. Strong contemporary gene flow between ecotypes also suggests isolation is either recent or insufficient to allow microsatellite loci divergence (Weese et al. 2012; Colborne et al. 2016). Complete isolation is also unlikely given numerous intermediate forms in both habitats (e.g., Jastrebski and Robinson 2004) and weak mate choice (Jarvis et al. 2017). Spatial assortative mating between habitats occurs and may be facilitated by matching habitat choice (Deleeuw 2021).

### Reproduction and early life history

Reproductive behaviour commences in late May when surface water reaches 20 °C (Amundrud et al. 1974; Keast 1980; Scott and Crossman 1998). Males construct and defend nest depressions typically in shallow bays and shorelines with soft substrates, and atypically in benthic depression at rocky pelagic shoals (Jastrebski and Robinson 2004; Colborne et al. 2016; McAllister et al. 2025). Females spawn in nests then leave while males care for and guard eggs over 11 to 21 days (mean 15 days; Danylchuk and Fox 1996). Embryos hatch 5–8 days post-spawn earlier in warmer water (Cargnelli and Neff 2006) and remain in the nest for up to 3 additional days until yolk sacs absorb and swim bladders inflate coincident with onset of exogenous feeding starting with small protists. At 8–11 days post-spawn, translucent larvae (4.5 mm Total Length or ‘TL’) feeding on small copepod nauplii in the water column leave the nest. Growing larvae consume increasingly larger zooplankton (Lemly and Dimmick 1982). The definitive juvenile phenotype develops at 5 weeks (~20 mm TL) and continues feeding on zooplankton throughout the natal summer (Keast 1980; Keast and Eadie 1984). As they grow, visual fish predation drives juveniles to refuge in benthic structure (Werner and Hall 1988) that can generate severe juvenile resource competition (Werner et al. 1983; Keast 1985; Osenberg et al. 1988, 1992). By end of natal summer juveniles are approximately 3% of adult body weight in this region (Keast and Eadie 1984). Earlier spawning increases mortality risk from cold events in early spring (Garvey et al. 2002) but when successful also increases late summer age-0 juvenile size (Cargnelli and Gross 1996). Body size has a positive effect on summer feeding and growth (Keast 1980, 1985) and survival (Cargnelli and Gross 1996; Garvey et al. 2002). Larger body size increases overwinter survival due to greater energy reserves and reduced metabolic costs (Bernard and Fox 1997; Cargnelli and Gross 1997; Shoup and Wahl 2011), and less over-winter predation (Shoup and Wahl 2008). In this region, sexual maturity typically occurs in the third summer (Fox 1994; Gillespie and Fox 2003).

### Study site—Ashby Lake

Samples were collected from Ashby Lake, Addington Highlands, Ontario, Canada (45.092N, 77.351W), a small (surface area 2.59 km^2^, maximum depth 36.6 m), post-glacial inland lake studied since 2000 (Jastrebski and Robinson 2004; Axelrod et al. 2022). Lake productivity is low (phosphorous concentration = 7.14 ug/L ± 3.89 sem; *n* = 16 years; mean summer secchi depth = 5.43 m ± 0.57 sem; *n* = 31 years; Ashby Lake Protective Association annual water quality records; https://www.ashbylake.net/). This population is representative of other polyphenic populations (Weese et al. 2012). Eleven submerged rocky shoals inhabited by pumpkinseed are separated from the shoreline by deeper water (>6 m depth) rarely traversed by adult pumpkinseed (Jarvis et al. 2020). Ancestral littoral habitat with soft organic substrates and vegetation is restricted to sheltered bays separated by segments of rocky shorelines with a narrow exposed littoral strip (<2 m deep, <5 m wide) that rapidly drops into deep water (Jarvis et al. 2021). Fish predators of juvenile pumpkinseed include smallmouth bass (*Micropterus dolomieu*) in all seasons and larger lake trout (*Salvelinus namaycush*) in cooler seasons (e.g., Blanchfield et al. 2023). No other centrarchid sunfish are present (Jastrebski and Robinson 2004).

### Sampling

We sampled zooplankton, other benthic reference prey and age-0 pumpkinseed weekly throughout the summer of 2014 from sites in three lake habitats: (1) sheltered littoral bays with soft sediments and macrophytes (ancestral habitat); (2) the deep open-water pelagic habitat (non-ancestral habitat), and (3) the narrow and exposed littoral shoreline habitat adjacent to deep waters, hereafter littoral, pelagic and shoreline, respectively. Shoreline habitat was included because spawning occurs there at low levels (Jarvis et al. 2021), and the shallow hard sediment, absence of macrophytes, exposure to wind and wave action and proximity to deep open water with zooplankton mix characteristics of the littoral and pelagic rock shoal habitats. Surface water temperature (~1 m depth) was monitored at two each of littoral and pelagic sites (submerged HoboTemp digital thermometers). Onset of nesting and spawning was determined by visual snorkel diver surveys twice per week at multiple sites from late May through mid-July.

### Zooplankton density

Zooplankton density was estimated from multiple horizontal surface plankton trawls (<2 m depth) per week between May 29th and August 1 st, from waters adjacent to littoral, pelagic and shoreline habitats (Table 1). Tows were grouped by month: June 31 st (‘June’ *n* = 13) and July (thereafter to August 1 st; n = 19). Two plankton nets (75 cm dia.; 500 micron mesh) were towed at 0.6 m/s or less at a measured velocity for 2 min (Keast 1980) and retained larger meso- and macroplankton, such as copepods, cladocerans and larger zooplankters. All three habitats were systematically sampled across daylight hours. Paired net contents were combined and half the sample preserved in 10% formaldehyde and half frozen for stable isotope analysis. Plankton were removed from the formaldehyde-preserved samples by filtering through paper, drying (40 °C for 48 h) to determine total dry mass. Zooplankton density was estimated as dry mg carbon per m^3^ of net-sampled water.

**Table 1:** Mean standard length (mm) and body mass (g), standard error of the mean, and sample sizes in parentheses of pumpkinseed larvae (June and July) and age-0 juveniles (August and September). Standard length is provided for each month in each lake habitat. Larval body mass could not be accurately measured, and so only juvenile mass is available in August and September

	Mean standard length (mm) ± sem Mean mass (g) ± sem (sample size)			
**Site type**	**June (larval)**	**July (larval)**	**August (juvenile)**	**September (juvenile)**
Littoral	4.73 ± 0.266	4.62 ± 0.246	18.87 ± 0.487	23.86 ± 0.254
	--	--	0.111 ± 0.008	0.241 ± 0.008
	(3)	(3)	(34)	(46)
Shoreline	4.15 ± 0.065	4.75 ± 0.269	18.03 ± 0.504	23.99 ± 0.376
	--	--	0.106 ± 0.011	0.229 ± 0.012
	(2)	(4)	(32)	(29)
Pelagic	4.48 ± 0.63	5.32 ± 0.249	22.23 ± 0.376	27.48 ± 0.355
	--	--	0.218 ± 0.011	0.325 ± 0.012
	(2)	(4)	(32)	(58)

### Larval and juvenile age-0 pumpkinseed sampling

Pumpkinseed larvae were sampled in June and July from the plankton trawls above. Larvae extracted from the formaldehyde-preserved and frozen plankton samples (littoral, pelagic and shoreline, above) under a dissecting microscope and stored in 90% ethanol (Formaldehyde/Frozen sample sizes: pelagic = 35/106 larvae; littoral = 26/306 larvae; shoreline = 235/122 larvae) were classified by site and date. Mean larval density per m^3^ water was estimated in each lake habitat from three to five plankton tows combined over two consecutive sampling days. Late season age-0 juveniles were collected in August and September using minnow traps and by snorkel diving with dip-nets at sites in each habitat (August 16 sample sizes: n_Littoral_ = 34; n_Pelagic_ = 32; n_Shoreline_ = 32; September 16 samples: n_Littoral_ = 52; n_Pelagic_ = 110; n_Shoreline_ = 32), and subsequently frozen.

### Estimating juvenile resource use with stable isotopes of C and N

**Fig. 1:**
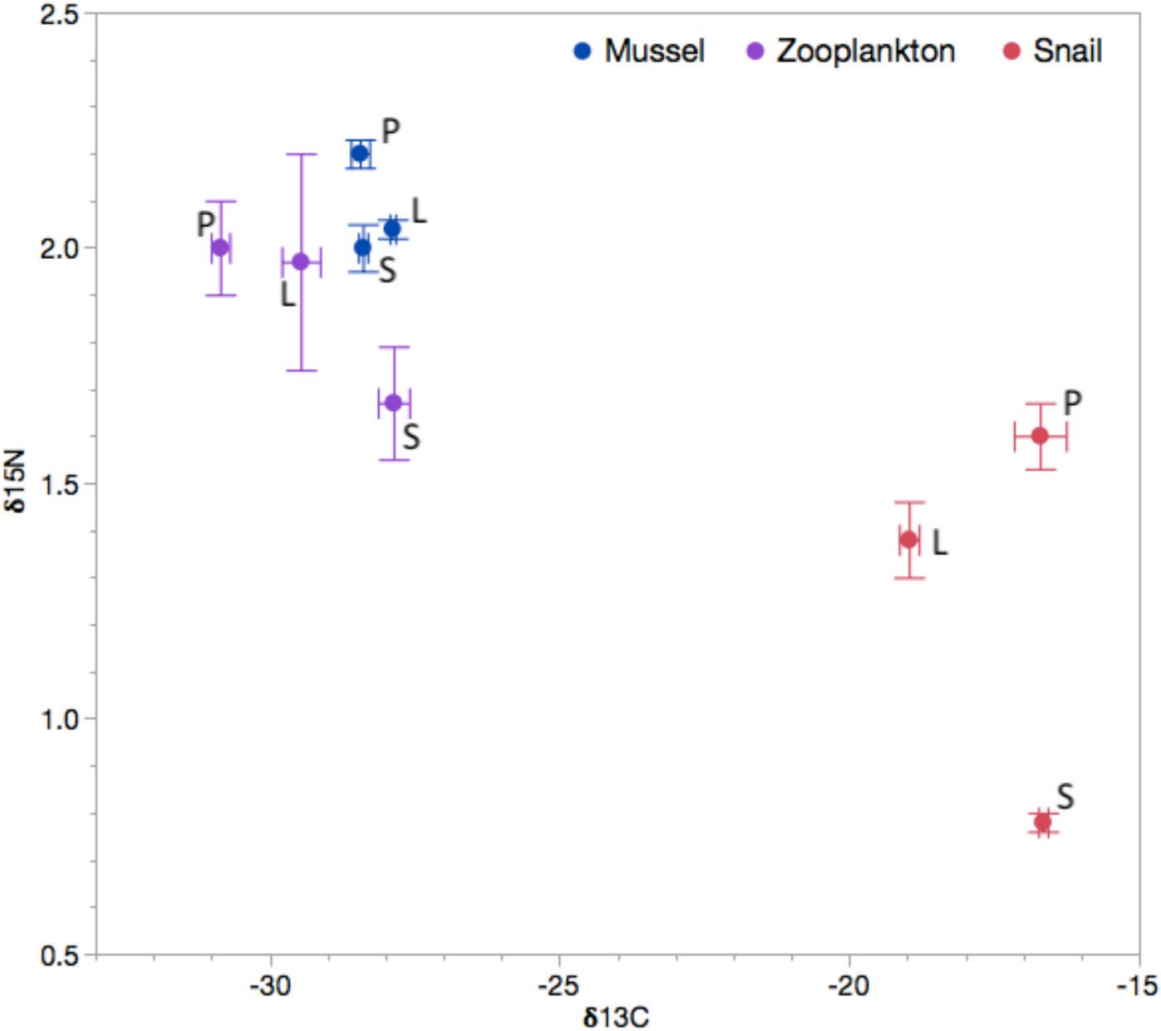
Plot of mean values of baseline prey resource samples from Ashby Lake in stable isotope space of δ^13^C and δ^15^N for 2014. Mussels (blue circles), mixed zooplankton (purple circles) and snails (red circles) are shown, with means (and standard errors) averaged over multiple samples taken from three habitats: pelagic (P), shoreline (S) and littoral (L)

Stable isotope analysis (SIA) of carbon (*δ*^13^C) and nitrogen (*δ*^15^N) of frozen larval and age-0 juveniles were compared against isotope values of reference prey that distinguish littoral (predominantly benthic algal-derived) and pelagic (predominantly phytoplankton-derived) energy pathways to estimate relative resource use from each pathway (e.g., Hecky and Hesslein 1995; Vander Zanden and Vadeboncoeur 2002; Vander Zanden et al. 2011). Reference samples were collected from all three habitats over the spawning period (Fig. 1). We first evaluated whether unionid mussels that feed primarily on phytoplankton at the base of the pelagic energy pathway reflect similar stable isotope ratios of zooplankton (Vander Zanden and Rasmussen 2001) by comparing mussel and zooplankton stable isotope ratios (separated from phytoplankton). Snails provide a similar representative baseline stable isotope ratio for benthic resources (Post 2002; Colborne et al. 2016). Reference samples were frozen on collection and the outer shells of snails and bivalves were removed. Multispecies zooplankton samples represent an overall zooplankton isotope measure reflecting the plankton-derived energy pathway. Independent spatial and temporal replicates of all reference taxa were maintained, while mussel or snails collected at the same time and location were combined to estimate stable isotopes. As expected, reference mussels and zooplankton each differed significantly from snails in their mean δ^15^N (Tukey HSD Mussels-Snails *t* = 5.2, *p* < 0.0001; Zooplankton-Snails *t* = -5.0, *p* < 0.0001) and δ^13^C (Mussels-Snails *t* = -24.6, *p* < 0.0001; Zooplankton-Snails *t* = 24.6, *p* < 0.0001) regardless of habitat, whereas mussels and zooplankton did not differ (δ^13^C Tukey HSD *t* = 2.1, *p* = 0.1; δ^15^N t = -0.2, *p* = 0.97). Despite fractionation differences between mussels and zooplankton, mussel tissue is a useful proxy of plankton-derived resource use because of the ten-fold greater difference between mussels and snails relative to that between mussels and zooplankton (Fig. 1). Furthermore, mussel tissues integrate pelagic primary production at a temporal scale more like that of snails that represent benthic productivity, whereas rapid plankton turnover likely generates a more variable signal.

Rapidly developing larvae and age-0 juveniles quickly integrate fluctuations in the isotopic composition of diet because rates of fish tissue turnover are proportional to body mass and growth rate (Fry and Arnold 1982; Herzka and Holt 2000; Post 2002). SIA was based on whole body preparations of larvae (Giraldo et al. 2011) and of age-0 pumpkinseed with the peritoneal cavity removed from larger individuals to remove any influence of gut contents on SIA (Feuchtmayr and Grey 2003). Samples were prepared by freeze drying (–50 °C for 24 h), grinding into a fine powder (ThermoSavant Fastprep grinder) and weighing to the nearest 0.001 mg to obtain a final sample weight of 0.4–0.6 mg. Dried individual larvae from similar temporal and spatial samples were combined because individuals weighed less than the minimum mass required to assess stable isotopes.

Quantification of sample stable isotope ratios was performed at the Trophic Ecology Lab at the Great Lakes Institute for Environmental Research (GLIER, University of Windsor, Windsor, ON, Canada) using a Costech Elemental Analyzer-Isotope coupled with a Thermo-Scientific Continuous Flow Ratio Mass Spectrometer (EA-IRMS, ThermoFisher Scientific, MA, USA). Stable isotope ratios were measured in per mil (‰) difference in heavy to light isotope ratio from a standard reference material (R_standard_) for N^15^:N^14^ (δ^15^N) and C^13^:C^12^ (δ^13^C), atmospheric nitrogen and Vienna Pee Dee Belemnite (VPDB), respectively:

$$dX=\left[Ratio_{sample}/Ratio_{s\tan dard}-1\right]$$

C^13^ is incorporated into fatty acids at lower rates compared to proteins and so biases δ^13^C values down in lipid rich tissues (Kiljunen et al. 2006). We corrected for any lipid effect in reference invertebrates following Fry et al. (2003) as adapted by Smyntek et al. (2007):

$$\delta^{13}C_{ex}=\delta^{13}C_{bulk}+6.3\left(\left(C:N_{bulk}-4.2\right)/C:N_{bulk}\right)$$

where δ^13^C_ex_ is the expected δ^13^C value of the reference taxon sample exclusive of lipids, δ^13^C_bulk_ is the observed δ^13^C value of a reference taxon sample, C:N_bulk_ is the observed atomic C:N ratio of a sample, and 6.3 and 4.2 are constants in the mass balance model (representing D, the mean ‰ discrimination factor between lipids and protein, and C:N_ex_, the average C:N ratio of lipid-extracted tissues from invertebrates).

Fish tissue samples were corrected using a lipid normalization model of Kiljunen et al. (2006):

$$\delta^{13}C_{ex}=\delta^{13}C_{bulk}+D(I+(3.90/1+287/L))$$

where,

$$L=93/\lbrack1+(0.246\times{(C:N_{bulk})\rbrack-0.775)}^{-1}\rbrack$$

L is the proportional lipid content of the sample, δ^13^C_ex_ is the expected, lipid-normalized value of a fish tissue sample, δ^13^C_bulk_ is the measured δ^13^C value of a fish tissue sample, C:N_bulk_ is the observed atomic C:N ratio of a sample, D is a constant (= 7.018) that is the isotopic difference between protein and lipid and the constant *I* = 0.048.

The percent contribution of planktonic-derived resources to the diet of larvae and juveniles was estimated using a two-end-member-mixing model (Post 2002) following Colborne et al. (2016):

$$\%\;Planktonic=\left(\delta^{13}C_{consumer}-\delta^{13}C_{base\;2}\right)/\left(\delta^{13}C_{base\;1}-\delta^{13}C_{base\;2}\right)\times100$$

where δ^13^C_consumer_ is the measured δ^13^C value for each juvenile or pooled larvae sample, δ^13^C_base 1_ is the mean δ^13^C measure of pelagic-sampled mussel and δ^13^C_base 2_ is the mean δ^13^C measure of littoral-sampled snail tissues. These baselines are the best estimates of isotopic conditions from benthic and planktonic energy pathways, respectively.

### Juvenile performance

Body size and condition were used to assess individual age-0 juvenile performance because these influence summer and winter season survival (Cargnelli and Gross 1996, 1997; Bernard and Fox 1997; Ludsin and DeVries 1997; Garvey et al. 2002; Shoup and Wahl 2008, 2011). Standard length (mm) was estimated from scaled digital images (Image-J digital image analysis software, Schneider et al. 2012) and blotted wet mass (g) to the nearest 0.1 mg. Juvenile condition was estimated by residual mass after linearly regressing mass against standard length using all samples combined. Age-0 status of juveniles was determined as the absence of annuli on each of five scales sampled from the side of a juvenile (Robinson et al. 1996) and assuming individuals were born in 2014. Assessing larval size was not possible due to processing for SIA above and the low precision at estimating preserved larval mass. We report standard length based on one larva sampled per site and day for all habitats combined over 2 months (n_June_ = 7; n_July_ = 11).

### Statistical analyses

Preliminary analyses used two-factor ‘general’ ANOVA models including an interaction to partition response variation between habitats (littoral, pelagic and shoreline), months (Larval: June and July; Juveniles: August and September) and their interaction. Response variables included zooplankton density (mg dry mass/m^3^ tow volume; value + 1 natural log-transformed); the tissue fraction arising from plankton-derived resources (% Planktonic; arcsine-transformed); and standard body length (mm) of post settlement juveniles. We followed up with detailed comparisons of responses among lake habitats in separate analyses by month. Heteroscedasticity among habitats was common and so we used a Kruskal-Wallis test followed by a Wilcoxon multiple comparisons (*⍺* < 0.05). To evaluate habitat effects on body condition, we compared the body mass of age-0 juveniles sampled in September using ANCOVA treating standard length as a covariate, followed by Tukey’s HSD tests to evaluate pair-wise habitat comparisons (*⍺* < 0.05). Analyses were performed with JMP Pro11 and we report 2-tailed *p*-values.

## Results

### Spawning and larval phenology

**Fig. 2:**
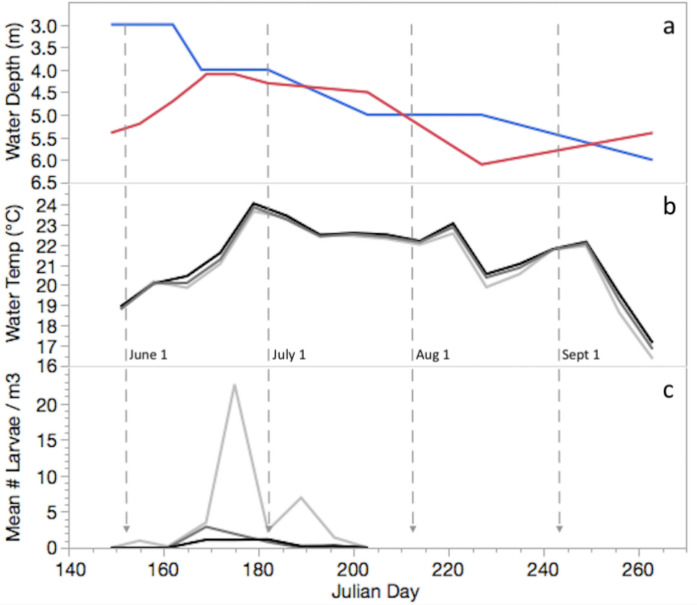
Summer season 2014 changes in, **a** depth (m) of Secchi reading (red) and Thermocline (blue) in the pelagic habitat. **b** Mean weekly water temperature at 1 m depth (°C; averaged from daily 6 am and 6 pm values from 2 to 3 sites per habitat). **c** Mean number of larvae per m^3^ of water volume (averaged over 3–5 plankton tows per habitat). Lake habitats in **b** and **c** are littoral (light grey), pelagic (black), and shoreline (dark grey)

Minor differences in the onset of nesting and spawning, and in free larval densities occurred between habitats in 2014. Male nesting commenced at littoral sites as surface waters reached 19.5 °C at the end of May (Fig. 2) and 10 days later at pelagic sites. Larvae first appeared June 3 in littoral trawls and June 17 in shoreline and pelagic trawls. Larval density peaked in late June in all habitats although density was seven times higher at littoral compared to shoreline or pelagic sites. A small second pulse of larvae occurred in early July for littoral trawls but not for shoreline or pelagic trawls. Larval density fell to zero by the third week of July, earlier in pelagic and shoreline compared to littoral sites.

### Zooplankton resource availability

**Fig. 3:**
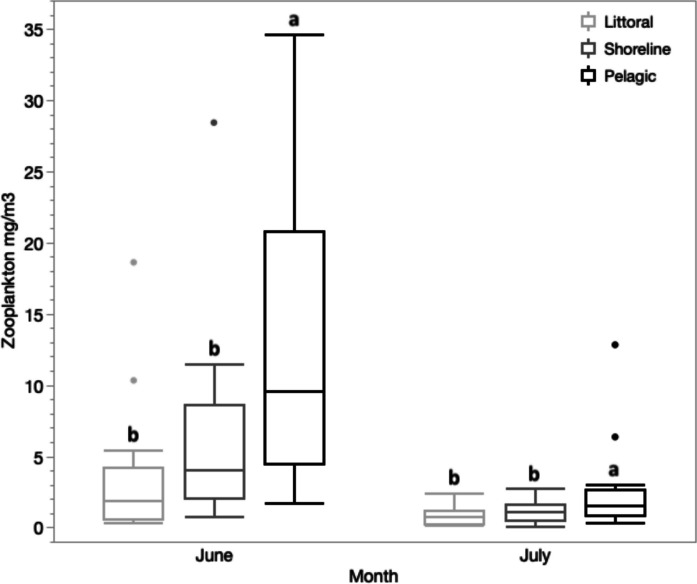
Boxplots of zooplankton abundance in June and July 2014 for three lake habitats: littoral (light grey), shoreline (dark grey) and pelagic (black). Boxes express the interquartile range (25th to 75th quantiles) surrounding the median (internal horizontal line). Whiskers extend ± 1.5× from their respective quartiles (interquartile range). Dots represent outliers. Letters above boxes reflect Wilcoxon multiple comparisons among habitats separately for each month. Trawl samples per habitat were *n* = 13 in June and *n* = 19 in July

Abundance of large zooplankton varied over summer months and among habitats (Fig. 3). The density of zooplankton declined by a factor of four from June to July (Month *F*_1,90_ = 61.8, *p* < 0.0001 Table S1; mean density, mg/m^3^: June = 4.73, July = 1.13) concurrent with increasing water transparency and a deepening thermocline (Fig. 2a). Temporal declines in zooplankton density were not influenced by habitat (Habitat-Month Interaction *F*_2,90_ = 2.3, *p* = 0.10). Zooplankton density over the spawning and larval periods was highest at pelagic sites (June: Habitat K-W *x*^2^ = 11.1, *p* = 0.004; July: K-W *x*^2^ = 11.0, *p* = 0.042). For each summer month, zooplankton density was generally greater in the pelagic compared to littoral and shoreline sites (Fig. 3).

### Larval diet

**Fig. 4:**
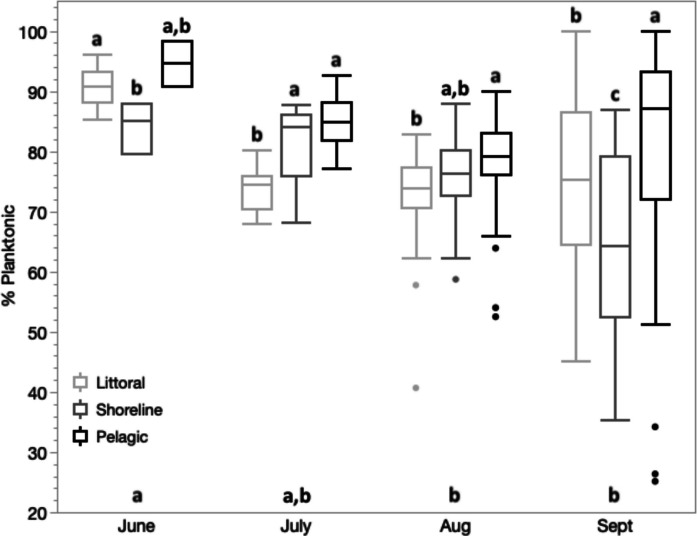
Boxplots of percent composition of age-0 pumpkinseed tissue derived from planktonic sources (%-Planktonic) in larvae (June and July) and juveniles (August and September) sampled from three lake habitats: pelagic (black), shoreline (dark grey) and littoral (light grey). Letters above boxes reflect Wilcoxon multiple comparisons among habitats separately for each month. Significant month effects are indicated by letters above the horizontal axis. Boxplot composition as in Fig. 3

The SIA analysis revealed that age-0 juveniles transitioned to using more benthic resources by the end of their natal summer as expected, although this transition was weakest in juveniles from pelagic sites (Fig. 4). The mean fish tissue fraction derived from planktonic sources (all habitats combined) declined in larvae sampled between June (90.6%) and July (80.3%), continuing to decline in juveniles in August (75.5%) thereafter remaining steady into September (76.6%) (Month *F*_3,276_ = 5.41, *p* = 0.001; Table S2). In each month, the rank order of median %-Plankton tissue values was always highest for pelagic juveniles, exceeding that of littoral fish except for June larvae (Fig. 4). However, the rank order of habitat differences shifted over months (Habitat-Month Interaction *F*_6,276_ = 3.25, *p* = 0.004). Multiple comparisons in each month revealed that at the start (June) and end (September) of summer, juveniles from shoreline sites had the lowest median fractions of %-Plankton, whereas in mid-summer (July and August) the lowest median fractions were expressed in littoral fish (Fig. 4). In contrast, the median %-Plankton tissue value in September was still 90% for pelagic juveniles, indicating less reliance on benthic resources compared to juveniles in the other two habitats.

### Age-0 growth performance

**Fig. 5:**
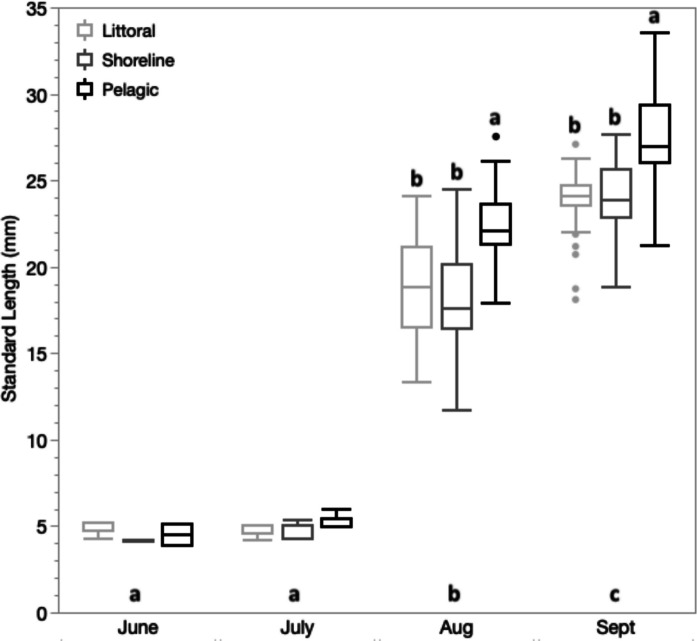
Boxplots of standard length of pumpkinseed larvae (June and July) and age-0 juveniles (August and September) sampled from three lake habitats: pelagic (black), shoreline (dark grey) and littoral (light grey). Letters above boxes reflect Wilcoxon multiple comparisons among habitats separately for each month in age-0 juveniles (larval sample sizes in June and July were inadequate to test habitat effects). Significant month effects are indicated by letters above the horizontal axis. Boxplot composition as in Fig. 3

**Fig. 6:**
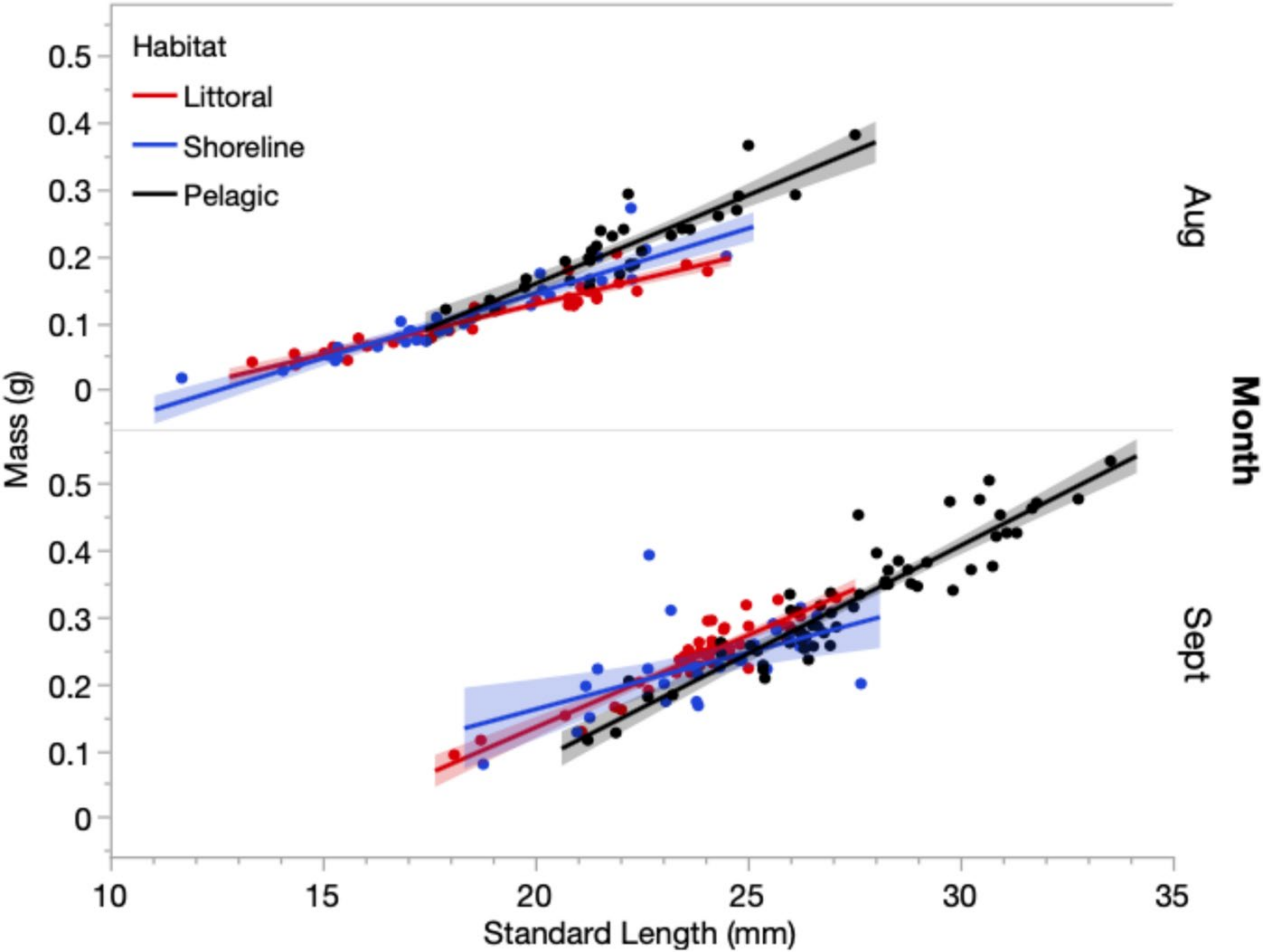
Differences in the body condition estimated as the linear relationship between mass and length for age-0 juvenile pumpkinseed between pelagic (black), exposed shoreline (blue) and littoral (red) habitats. Slopes were significantly steeper for pelagic juveniles in each month. The confidence region for the line of best fit is shown

By summer’s end habitat differences in the availability and utilization of zooplankton resources corresponded to mean differences in juvenile body size (Fig. 5) and body condition (Fig. 6). Body length increased over months in each of larval and age-0 juvenile stages (Table S3), but pelagic individuals were on average longer than either littoral or shoreline individuals in both August (K-W *x*^2^ = 31.7, *p* < 0.0001) and September (K-W *x*^2^ = 55.7, *p* < 0.0001; Fig. 5). Furthermore, variation in length among individuals was significantly greater at pelagic than at littoral sites in September (Bartlett *F* = 5.2; *p* = 0.006) but not in August (*p* = 0.2).

Body condition estimated as residual mass at length was also higher in age-0 pelagic juveniles in August, reflecting a steeper mass allometry with length (ANCOVA Standard Length–Habitat interaction *F*_2,92_ = 11.7, *p* < 0.0001; Fig. 6; Table S4). However, by September there was no difference in the size allometries of age-0 juveniles between littoral and pelagic habitats, whereas mass allometry was shallower in shoreline juveniles (Table S5). Littoral juveniles were shorter than pelagic juveniles in September but also 10.5% heavier than comparable small-size pelagic juveniles (ANCOVA Habitat *F*_1,101_ = 14.4, *p* < 0.0003; adj. mean and SE littoral = 0.304 g ± 0.0052; pelagic = 0.275 g ± 0.0045).

## Discussion

By providing ecological opportunities, habitat shifts are thought to initiate fish diversification (e.g., Siwertsson et al. 2010, 2013). We explored whether pelagic zooplankton represent an ecological opportunity for age-0 juvenile pumpkinseeds spawned on rocky shoals by comparing diet and growth performance with local juveniles in the ancestral littoral habitat. Pelagic juveniles received nutritional and growth advantages over their first summer despite a shorter summer growth period due to delayed spawning compared to littoral sites. By summer’s end, pelagic juveniles were on average 15% longer and 35% heavier than those at local littoral and shoreline sites. Juvenile growth was associated with local zooplankton density across habitats. Planktonic sources contributed 91% to larval tissue at the start of independent life stages in June, and this proportion declined to 76% by late summer, indicating increased use of benthic resources over the natal summer. However, the median decline was least for pelagic juveniles, indicating that a greater or longer reliance on zooplankton resources was related to the growth benefit. This indicates that zooplankton resources provide an ecological opportunity to early life-stage juveniles spawned on pelagic shoals as well as to adults there.

It is possible that pumpkinseed at pelagic sites are maintained by ongoing immigration from local littoral sites since their initial colonization. However, we think an ecological sink population structure at pelagic sites is unrealistic for several reasons. First, pumpkinseed annually spawn and produce viable larvae on pelagic shoals (Jastrebski and Robinson 2004; Colborne et al. 2016) indicating viable reproduction. Second, age-0 juveniles that develop on pelagic shoals rely more on pelagic zooplankton and grow better than juveniles in the ancestral littoral habitat over their natal summer, demonstrating improved early-life performance under non-ancestral conditions. Interestingly, juveniles in the shoreline habitat did not gain a similar benefit despite access to pelagic zooplankton, suggesting local recruitment may be weaker there. Third, multiple size and age classes of juveniles are consistently present on pelagic shoals, suggesting persistent successful recruitment (Robinson, Unpub. data). Fourth, adult pumpkinseed rarely seem to make long distance movements between lake habitats (McCairns and Fox 2004; Jarvis et al. 2020) and when they do they seem to select habitats based on phenotype (Deleeuw 2021). In the absence of local recruitment, strong immigration from other sites would be required to maintain the high local density of pumpkinseed at pelagic sites (Jarvis et al. 2020). Fifth, individual habitat specialization and preference likely promotes local recruitment. Plastic developmental responses to pelagic cues improve juvenile feeding performance on zooplankton (Parsons and Robinson 2007), an important but atypical component of the adult pelagic diet (Jastrebski and Robinson 2004; Weese et al. 2012; Colborne et al. 2016). Furthermore, Deleeuw (2021) demonstrated adult matching habitat choice (Edelaar et al. 2008; Bolnick et al. 2009) that if present in juveniles would also favour local recruitment (Turko and Rossi 2022). Taken together, these observations suggest that pelagic shoals in Ashby Lake may provide the conditions that demographically favour a local subpopulation.

Recruitment at pelagic shoals is unlikely exclusively derived from local reproduction because allelic variation is not spatially structured between habitats here (Weese et al. 2012; Colborne et al. 2016). How are littoral and pelagic sites demographically connected? Dispersal may be more likely for larvae than for juveniles or adult pumpkinseed. Fish larvae typically have little capacity for directed swimming over long distances but can be passively transported in surface water moved by sustained winds and tides (e.g., Brodnik et al. 2016). Wind-driven water movements are sporadically strong in Ashby Lake during the summer, especially at exposed pelagic and shoreline sites (Robinson Pers. obs.). However, high condition fish larvae can also resist horizontal transport via short vertical movements (Sclafani et al. 1993; Paris and Cowen 2004; Jones et al. 2005). Thus, the generally larger larvae at pelagic sites here may have some capacity to avoid transport. Opposite transport from sheltered littoral bays to pelagic shoals is possible, especially since the larger ancestral littoral habitat likely produces more total larvae overall in Ashby Lake. However, bulk water movements from sheltered bays are also less likely and larvae may also resist movement by refuging in nearshore structure (Bertolo et al. 2012). Evaluating the behaviour and dispersal of fish larvae from different lake habitats is critical to assess how connectivity influences population structure here.

Larger juveniles and adults have considerable capacity for independent movement but may not move long distances for several reasons. Juvenile dispersal across habitats is less likely because of exposure to predation risk. For example, centrarchid sunfish below 80 mm TL largely avoid travelling because of predation from smallmouth bass during movements away from benthic refuge (e.g., Werner et al. 1983; Mittelbach 1984; Werner and Hall 1988). Larger pumpkinseeds do not face this risk but nevertheless express considerable site or habitat fidelity (McCairns and Fox 2004) and rarely move between habitats (Jarvis et al. 2020), although the reason why is not known. Adults that do move exhibit matching-habitat choice behaviour (Edelaar et al. 2008) that associates phenotypes with habitats and so reduces connectivity across habitats here (Deleeuw 2021). It is not clear when habitat selection behaviour develops in pumpkinseed, but plausibly quite early given plastic developmental responses cued by diet and predator cues in juveniles (Parsons and Robinson 2006; Januszkiewicz and Robinson 2007; Axelrod et al. 2022). Coupled with the development of habitat selection behaviour, functional plastic responses provide a developmental mechanism for individual specialization (Verzijden and Cate 2007; Stolz 2017; Turko and Rossi 2022) that could favour local retention. However, if larvae disperse between habitats before they settle, then individual specialization that develops after arrival could contribute to recruitment increasing connectivity across habitats. The demographic effects of larval dispersal are largely unknown in most polyphenic fish populations.

Juvenile mortality also has a strong effect on recruitment (Miller et al. 1988; Houde 1994), and the factors that regulate overwinter mortality are poorly understood in most fishes in northern lakes. Juvenile body size regulates over-winter mortality due to better energy reserves and reduced metabolic demands in winter when feeding largely ceases (Keast 1968; Ludsin and DeVries 1997; Cargnelli and Gross 1997). The threshold age-0 body size for winter survival increases with longer winters found at higher latitude. For example, the age-0 pumpkinseed size threshold increases from 26 mm total length in southerly Lake Ontario (Murphy et al. 2012) to 35 mm in more northerly inland lake populations like Ashby Lake (Fox and Keast 1991; Garvey et al. 2002). We observed most pelagic age-0 fish approaching 35 mm total length in September while littoral and shoreline age-0 pumpkinseed were notably smaller here, suggesting overwinter mortality risk may paradoxically be greater in the ancestral littoral habitat as found in some ponds (Fox and Keast 1991). However, these size thresholds should be interpreted cautiously because they ignore potential habitat effects and annual variation (e.g., Svanbäck and Persson 2004). The demographic effects of overwinter juvenile mortality in northern lake populations require further study.

In addition to overwinter mortality, predation and competition may also influence local recruitment (Meyers et al. 1997; Byström et al. 2004; Ward et al. 2006). Smallmouth bass, a key juvenile predator, are present in all three Ashby Lake habitats and benthic refuges are used in all habitats here (Robinson pers. obs.) but rates of predation are not available for comparison. However, refuging juveniles can face severe competition over benthic resources at least under littoral conditions (Mittelbach 1988; Fox 1994), but how this may vary among habitats in Ashby Lake is also not known. Competition among juveniles at littoral sites is plausible given the higher density of pumpkinseed larvae and the greater seasonal decline in zooplankton at littoral compared to pelagic sites. At pelagic sites benthic prey are less common but zooplankton provide a replaceable resource. Whether refuging juvenile pumpkinseed there experience competition with each other or from the high density of adult pumpkinseed (Jarvis et al. 2020) that also consume pelagic zooplankton is not clear. On the one hand, the loss of superior body condition for age-0 pelagic juveniles from August to September and greater size variation of late season juveniles at pelagic compared to littoral sites is consistent with increased competition (Svanbäck and Bolnick 2007). On the other hand, any competitive effects may be relatively minor because age-0 pelagic juveniles were on average larger than those in the other two habitats. Lastly, we caution that equilibrium assumptions of population dynamics may not apply for all trophic polyphenisms. Resources can fluctuate in time due to extrinsic habitat conditions or intrinsic consumer population dynamics (Svanbäck and Persson 2004).

Several factors may have influenced our estimates of seasonal growth. We can rule out temperature dependent metabolic effects on September body size because surface water temperature differences were minimal between habitats. Similarly, differences in timing of spawning cannot explain habitat differences in juvenile growth (Cargnelli and Gross 1997; Murphy et al. 2012). Spawning commenced earliest at littoral sites and so could not have yielded superior growth in pelagic juveniles. However, two other factors may lead us to over-estimate age-0 growth in pelagic juveniles here. The first is local adaptation in the form of juvenile specialization at pelagic sites. Genetic differences identified through common-garden studies (Parsons and Robinson 2006; Januszkiewicz and Robinson 2007; Axelrod et al. 2022) along with trade-offs in performance across prey and habitats (Robinson et al. 1996; Parsons and Robinson 2007) suggest some local adaptation is possible. Genomic differentiation between ecotypes is possible with gene flow (e.g., Ford et al. 2015) although this may be less common for trophic traits than for mate preference traits (Malinsky et al. 2015) which are weak here (Jarvis et al. 2017). Second, estimates of mean growth may be biased by any differences in juvenile survival between habitats. In fish, size frequently influences age-specific survival. If juvenile mortality were greater overall at pelagic sites and size influenced survival, then mean growth estimates based on survivors will be biased upwards (Heath and Gallego 1997). Again, age- and habitat specific mortality are required to better understand this risk.

During adaptative radiation selection acting between viable populations under different conditions drives the formation of new species. Habitat shifts are a key process that by providing ecological opportunities allow viable populations to establish under different conditions. Whether a population is viable in a non-ancestral habitat depends on local reproduction and juvenile performance. A habitat that provides benefits to juvenile stages likely increases the viability of a local subpopulation by permitting local recruitment in contrast to one that limits reproductive success and juvenile performance. We report on successful reproduction and strong age-0 juvenile growth under non-ancestral conditions that are not consistent with an ecological sink model for pelagic pumpkinseed. By increasing the probability that individuals spawned under non-ancestral conditions persist through early life stages, juvenile performance may contribute to sustained local recruitment. This, in turn, may maintain a locally viable pelagic subpopulation that provides the demographic context in which plastic developmental responses that enhance individual performance, and habitat preferences form, and where selection can potentially operate over development to promote ecological and phenotypic diversification (e.g., Wimberger 1994; Skúlason et al. 2019; Turko and Rossi 2022). Polyphenism in centrarchid and other fishes in low diversity lakes are viewed as arising from ecological opportunities due to habitat shifts among pelagic, littoral and benthic habitats to generate functionally novel phenotypes under non-ancestral conditions. Yet the demographic consequence of habitat shifts remain largely unexplored for most resource polyphenisms and polymorphisms (Johannesson et al. 2025). The question remains — after a habitat shift, what factors that regulate population demography, dynamics and connectivity will favour adaptive divergence?

## Supplementary Information

Below is the link to the electronic supplementary material.

ESM1 (DOCX 16.9 KB)

## Data Availability

Data used in this manuscript is available through the University of Guelph research data repositories part of the Borrealis V1 (the Canadian Dataverse Repository/le dépôt Dataverse Canadien) as the file: *Supplemental data for, Effects of spawning habitat on the performance of age-0 pumpkinseed sunfish (Lepomis gibbosus) in a Canadian shield lake*. 10.5683/SP3/DIJ6XF.
