# Supplementary material for: Effects of spawning habitat on the performance of age-0 pumpkinseed sunfish (Lepomis gibbosus) ecotypes in a Canadian shield lake
Source: Environ Biol Fishes. 2026 Mar 28;109(2):65. doi: 10.1007/s10641-026-01836-6 (PMC13033004; doi:10.1007/s10641-026-01836-6)
Supplement: Supplementary file 1 — (DOCX 16.9 KB) [file 10641_2026_1836_MOESM1_ESM.docx]

**Supplementary Materials**

**General statistical model summaries**

| Term | *F* | *p* | *df* |  |
| --- | --- | --- | --- | --- |
| Habitat | 15.4 | <0.0001 | 2 |  |
| Month | 61.8 | <0.0001 | 1 |  |
| Interaction | 2.4 | 0.098 | 2 |  |
|  | **R^2^ adj.** | ***F*** | ***p*** | **df** |
| Model stats. | 0.484 | 18.8 | <0.0001 | 5, 90 |

S1. Summary of ANOVA model results evaluating the influence of Habitat, Month and their interaction on zooplankton density (natural log transformed).

| Term | *F* | *p* | *df* |  |
| --- | --- | --- | --- | --- |
| Habitat | 0.97 | 0.38 | 2 |  |
| Month | 5.41 | 0.001 | 3 |  |
| Interaction | 3.25 | 0.004 | 6 |  |
|  | **R^2^ adj.** | ***F*** | ***p*** | **df** |
| Model stats. | 0.154 | 5.74 | <0.0001 | 11, 276 |

S2. Summary of ANOVA model results evaluating the influence of Habitat, Month and their interaction on percent contribution to age-0 pumpkinseed tissue of planktonic derived energy and nutrients (arcsine-square root transformed).

| Term | *F* | *p* | *df* |  |
| --- | --- | --- | --- | --- |
| Habitat | 0.04 | 0.96 | 2 |  |
| Month | 419.2 | <0.0001 | 3 |  |
| Interaction | 1.48 | 0.19 | 6 |  |
|  | **R^2^ adj.** | ***F*** | ***p*** | **df** |
| Model stats. | 0.858 | 137.4 | <0.0001 | 11, 238 |

S3. Summary of ANOVA model results evaluating the influence of Habitat, Month and their interaction on values of age-0 pumpkinseed standard length.

| Term | Estimate | Std. Error | *t* | *p* |
| --- | --- | --- | --- | --- |
| Intercept | -0.264 | 0.019 | -13.97 | <0.0001 |
| Standard length | 0.020 | 0.001 | 22.08 | <0.0001 |
| Habitat (Lit) | -0.014 | 0.004 | -3.74 | 0.0003 |
| Habitat (Shr) | 0.001 | 0.004 | 0.24 | 0.81 |
| Habitat (Lit)*SL-19.7 | -0.005 | 0.001 | -4.29 | <0.0001 |
| Habitat (Shr)*SL-19.7 | -0.001 | 0.001 | -0.73 | 0.46 |
|  | **R^2^ adj.** | ***F*** | ***p*** | **df** |
| Model stats. | 0.915 | 198.5 | <<0.0001 | 5, 92 |

S4. Summary of ANCOVA model results evaluating the influence of Standard length (mm), Habitat and their interaction on juvenile body mass (g) for age-0 juvenile samples from August.

| Term | Estimate | Std. Error | *t* | *p* |
| --- | --- | --- | --- | --- |
| Intercept | -0.388 | 0.041 | -9.59 | <0.0001 |
| Standard length | 0.026 | 0.002 | 15.55 | <0.0001 |
| Habitat (Lit) | 0.019 | 0.006 | 3.12 | 0.0023 |
| Habitat (Shr) | -0.012 | 0.006 | -1.84 | 0.068 |
| Habitat (Lit)*SL-25.5 | 0.002 | 0.003 | 0.78 | 0.44 |
| Habitat (Shr)*SL-25.5 | -0.009 | 0.003 | -3.38 | 0.001 |
|  | **R^2^ adj.** | ***F*** | ***p*** | **df** |
| Model stats. | 0.825 | 125.9 | <<0.0001 | 5, 127 |

S5. Summary of ANCOVA model results evaluating the influence of Standard length (mm), Habitat and their interaction on juvenile body mass (g) for age-0 juvenile samples from September.
